# Supplementary material for: The stigma of alcohol use during pregnancy: Exploring the impact of alcohol strength and social context on public perceptions
Source: PLoS One. 2026 Jan 8;21(1):e0339954. doi: 10.1371/journal.pone.0339954 (PMC12782365; doi:10.1371/journal.pone.0339954)
Supplement: S1 File — (DOCX) [file pone.0339954.s001.docx]

**Supplementary materials**

**Recruitment details for Prolific:** No participants were recruited through Prolific for study 1, all of participants were recruited on Prolific for study 2 and 3. Participants were remunerated with either university course credits (n=179) or ~£6 per hour (n=875).

**Example Vignette:**

Sarah was attending a friend’s wedding. The service has finished, and all the guests have arrived at the reception. It’s a warm, sunny day and the reception is taking place in the grounds of a hotel. There is a large white marquee, set up with tables and a dance floor. Music is playing in the background, the atmosphere is happy and everyone is smiling.

Sarah is talking with friends, some she sees regularly and others she haven't seen for a while. Sarah enjoys reminiscing and catching up on what people are doing with their lives. Everyone is laughing and Sarah is really enjoying yourself. Sarah feels happy and excited. She decides to go to the bar and get a drink.

They all walk over to the bar and look at what’s available, before everyone starts to talk about what drink everyone wants. Everyone decides to have a glass of sparkling wine to celebrate. Sarah really wants to join her friends in having a glass of wine. Sarah is pregnant.

Sarah looks at what’s available and ask the bar person if there’s an alcohol-free sparkling wine. There is a no-alcohol sparkling wine available. Sarah decides to have a glass of alcohol-free wine and enjoy the special occasion with her friends
