# Supplementary material for: The stigma of alcohol use during pregnancy: Exploring the impact of alcohol strength and social context on public perceptions
Source: PLoS One. 2026 Jan 8;21(1):e0339954. doi: 10.1371/journal.pone.0339954 (PMC12782365; doi:10.1371/journal.pone.0339954)
Supplement: Attachment — Submitted filename: Number PONE-D-25-19244-2025.docx [file pone.0339954.s002.docx]

Thank you for taking time to work on this manuscript. It is an important topic that can be contribute to the wellbeing of maternal health. Below are a few comments that I feel can make your manuscript strong.

Abstract

1. The way you have started the introduction of your abstract seems lacking. For example, you do not state what the problem is and why it is important but you dive straight to examining a problem that is not well articulated. Please introduce your study well
2. In the methodology section, you do not state precisely how your data was analysed and the software used. Please review and make it clear.
3. I assume these are your results: Pregnant women consuming standard and low-alcohol drinks were more stigmatised than those consuming alcohol-free beverages across multiple aspects of stigma. Stigma attribution also varied between low and standard alcohol consumption conditions yet were inconsistent. You have not guided the reader to follow how consuming alcohol was more stigmatizing and also how attribution varied. This would benefit the reader if you drew some statistical expressions of this rather than just writing a test that is not well substantiated. This needs to be rewritten and present the results well.
4. What is the implication of your study/ its contribution to policy and practice? I think the most important question to answer her is so what from what you have presented.

Main body

Introduction:

5. In the second paragraph of the introduction, you state: In order to develop more effective, tailored alcohol interventions to reduce alcohol exposed pregnancy, it is also important to understand why women do not drink and why they do not seek help for alcohol use during pregnancy. This statement is confusing. Are you trying to explore why pregnant women do not drink alcohol or why they drink? Please review this and make it clear on what you are trying to bring to the attention of the reader.
6. You state standard alcohol drinks but you have not defined what this means and even give some examples for people to understand.
7. In the introduction, there is where you mention “NoLo drinks”. Please define this in the context of the presentation so that people can associate with it.

Methods:

8. Where was this study conducted? Please state the date of data collection.
9. How was data collected and who collected it? Was it a self-administered questionnaire or there was somebody who was taking the participant through? This is not clearly stated and needs to be made clear.
10. Were your participants only women? If so, do you think that you missed important views from the men that would have informed your study? I realise in your demographics, you had around 11. Please give reasons why this sample is so small and basically the rationale for these 11.
11. Best practice is to define an abbreviation the first time you use it. For example answer the SSA and PPSM these terms are first presented as abbreviations and later put in full down. Please rectify this.
12. Stigma and Attribution Assessment (SSA): is this a conventionally accepted abbreviation? If not, why not SAA? You will need to explain this so that we are on the same page.

Results section

13. In study 2, you state: Consumption of standard alcohol drinks were consistently stigmatised more than those consuming alcohol-free drinks (p’s<.001). What is new about this? It is common knowledge that those who drink alcohol are more criticized than those who do not drink alcohol. May be you need to throw some light on this. What were some of the reasons that were advanced for this result?
14. In study 3, you talk about types of alcohol but these are not mentioned anywhere. I think your results would be much stronger if you gave some examples of the types and why more people probably drink more of one type than the other. What was the favourite type among pregnant women?

Discussion

15. In this section, I would think that you state your main finding and its implication on policy and practice and then proceed to give a thorough discussion of your results giving special consideration to the main findings in the results. Please double check and see whether this is consistent.
16. Do you think that having carried out only a quantitative component was a limitation? This is because we miss to see the voices of people explaining why the numbers look the way they are now.
